# Longitudinal Relationship between Self-efficacy and Posttraumatic Stress Symptoms 8 Years after a Violent Assault: An Autoregressive Cross-Lagged Model

**DOI:** 10.3389/fpsyg.2017.00913

**Published:** 2017-06-01

**Authors:** Egil Nygaard, Venke A. Johansen, Johan Siqveland, Ajmal Hussain, Trond Heir

**Affiliations:** ^1^Department of Psychology, University of OsloOslo, Norway; ^2^Resource Centre on Violence, Traumatic Stress and Suicide Prevention, Western Norway (RVTS West), Haukeland University HospitalBergen, Norway; ^3^Western Norway University of Applied SciencesBergen, Norway; ^4^Department of Mental Health Services, Akershus University HospitalLørenskog, Norway; ^5^Institute of Clinical Medicine, University of OsloOslo, Norway; ^6^Norwegian Centre for Violence and Traumatic Stress StudiesOslo, Norway

**Keywords:** assault, autoregressive cross-lagged, longitudinal, posttraumatic stress symptoms, PTSD, self-efficacy

## Abstract

Self-efficacy is assumed to promote posttraumatic adaption, and several cross-sectional studies support this notion. However, there is a lack of prospective longitudinal studies to further illuminate the temporal relationship between self-efficacy and posttraumatic stress symptoms. Thus, an important unresolved research question is whether posttraumatic stress disorder (PTSD) symptoms affect the level of self-efficacy or vice versa or whether they mutually influence each other. The present prospective longitudinal study investigated the reciprocal relationship between general self-efficacy (GSE) and posttraumatic stress symptoms in 143 physical assault victims. We used an autoregressive cross-lagged model across four assessment waves: within 4 months after the assault (T1) and then 3 months (T2), 12 months (T3) and 8 years (T4) after the first assessment. Stress symptoms at T1 and T2 predicted subsequent self-efficacy, while self-efficacy at T1 and T2 was not related to subsequent stress symptoms. These relationships were reversed after T3; higher levels of self-efficacy at T3 predicted lower levels of posttraumatic stress symptoms at T4, while posttraumatic tress symptoms at T3 did not predict self-efficacy at T4. In conclusion, posttraumatic stress symptoms may have a deteriorating effect on self-efficacy in the early phase after physical assault, whereas self-efficacy may promote recovery from posttraumatic stress symptoms over the long term.

## Introduction

Victims of violent physical assault may experience lasting posttraumatic stress symptoms that are sometimes so severe that they meet the diagnostic criteria for PTSD (APA, 2013). In previous articles examining the present sample, the prevalence of probable PTSD was 31% 1 year after a violent assault (Johansen et al., 2007) and 19% after 8 years (Johansen et al., 2013). While many PTSD risk factors (e.g., type and severity of trauma, being female, low socioeconomic status and pre-trauma mental health problems) are well documented (Norris et al., 2002; Ozer et al., 2003), less is known about protective factors. One protective factor may be self-efficacy, that is, “an individual’s belief in their ability to manage their symptoms to unexpected events and to produce desired effects in a given activity” (Bandura, 1997). Self-efficacy is assumed to reduce posttraumatic stress symptoms through engagement in constructive regulation of cognitive, motivational, affective, and decisional processes (Bandura, 1997; Simmen-Janevska et al., 2012). Individuals with high self-efficacy consider anxiety and stress symptoms as controllable and temporary (Cervone, 2000; Leganger et al., 2000). The main aim of this study was to investigate the long-term reciprocal relationship between posttraumatic stress symptoms and general self-efficacy (GSE) after an assault.

Previous studies of posttraumatic stress symptoms and self-efficacy have primarily reported that high self-efficacy is related to lower levels of posttraumatic stress symptoms (Scholz et al., 2002; Luszczynska et al., 2009). If high self-efficacy indeed decreases the severity of posttraumatic stress symptoms, interventions promoting self-efficacy could be helpful for assault victims. However, an important caveat is that most previous research studies are cross-sectional (Luszczynska et al., 2009; Simmen-Janevska et al., 2012). The few existing longitudinal studies have produced mixed findings concerning the temporal relationship between self-efficacy and posttraumatic stress symptoms. Some longitudinal studies of natural disaster survivors found self-efficacy to be related to lower levels of posttraumatic stress symptoms longitudinally (Benight et al., 1999; Benight and Harper, 2002) or to moderate the effect of social support on stress symptoms (Warner et al., 2015). However, we previously found self-efficacy to be related only to concurrent posttraumatic stress symptoms (Johansen et al., 2007) and to be unrelated to posttraumatic stress symptoms beyond 6 months post-disaster (Nygaard et al., 2016). To our knowledge, the only prospective study concerning the temporal relationship between self-efficacy and posttraumatic stress symptoms used three assessments over an 8-month period and found that CSE was significantly related to later reductions in PTSD symptoms, while PTSD symptoms did not predict later reductions in CSE (Bosmans and van der Velden, 2015). Thus, an important unresolved research question is whether PTSD symptoms affect the level of self-efficacy or vice versa or whether they mutually influence each other.

The main aim of this study was to investigate the long-term reciprocal relationship between posttraumatic stress symptoms and GSE after physical assault by investigating the temporal relationship between self-efficacy and posttraumatic stress symptoms in assault victims over an 8-year period. Such a long period is essential because assault victims may experience posttraumatic stress symptoms for an extended period of time (Shalev, 2001; Marshall et al., 2010; Johansen et al., 2013). We hypothesized that higher self-efficacy would promote recovery and be related to the reduced severity of subsequent posttraumatic stress symptoms. A secondary aim was to investigate the stability of GSE over time, which we expected, based on previous studies, to be high, but not high enough to qualify as having trait-like qualities.

## Materials and Methods

### Design

**FIGURE 1:**
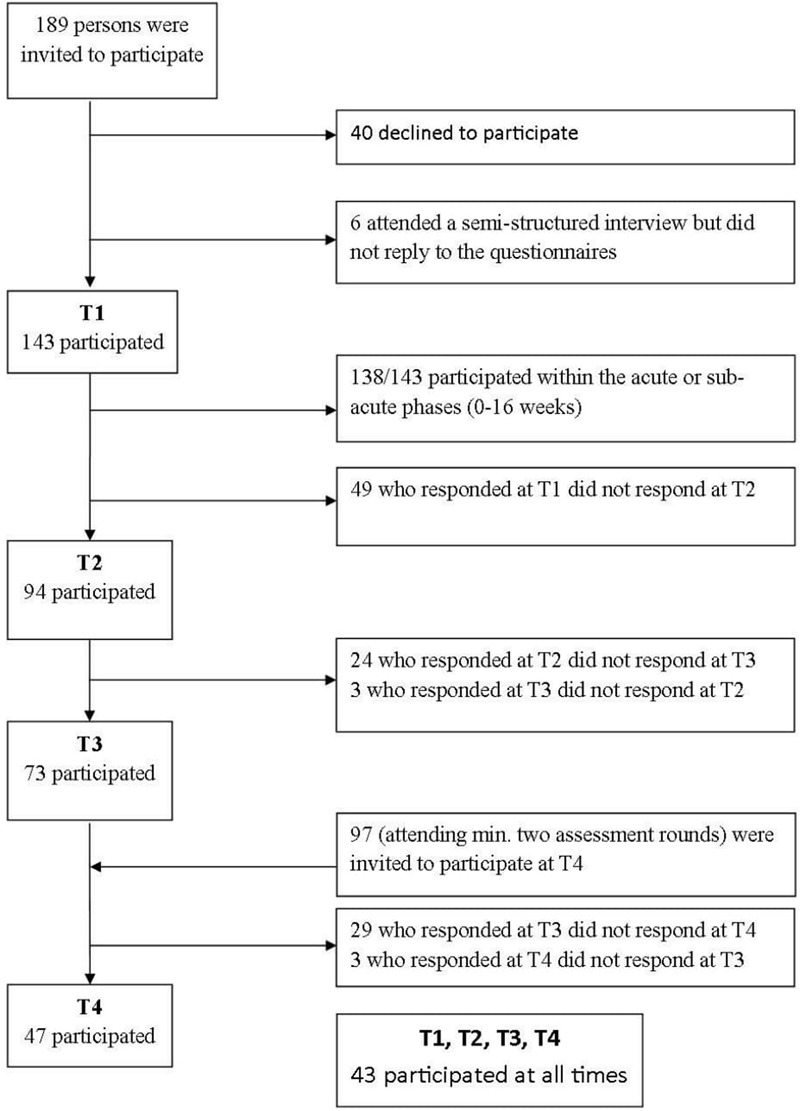
Flowchart for inclusion of participants.

The present study had a one-group prospective design with 143 physical assault victims. Here, we report the data from four self-reported assessments collected over a period of 8 years combined with the results of a semi-structured interview performed at the first assessment. Most of the respondents (138/143, 97%) completed the initial assessment (T1) between a few days and 16 weeks after the assault, while 5 (3%) completed the assessment more than 16 weeks post-assault. The participants thereafter participated in assessments at 3 months (T2), 12 months (T3), and 8 years (T4) after the first assessment. The flow chart in **Figure 1** presents further information about the recruitment procedure.

### Participants and Ethical Approval

All adults receiving care from a medical emergency unit or submitting a police report after being physically assaulted by someone other than a family member or former intimate partner were eligible for inclusion. Potential participants were recruited with the assistance of local police and medical services in the communities of Bergen and Oslo, Norway. All of the participants signed informed consent forms and the study was approved by the regional committee for medical research ethics, West (REK-West, no. 154.01) and by the Privacy Ombudsman, Norwegian Social Science Data Services (NSD, no. 8750). The second author (VJ) conducted all the interviews.

At T1, there were 143 participants, and the response rates were 66% (*n* = 94) at T2 and 51% (*n* = 73) at T3. Participants attending at least two out of the first three assessments (*n* = 97) were invited to participate at T4; the response rate at T4 was 48% (*n* = 47), or 33% of the original sample (**Figure 1**).

The majority of the victims were assaulted in a public place (88%) by an unknown perpetrator (93%). At T1, the mean age of the participants was 30.6 years (*SD* = 11.1), and 80% were male. All participants, except one, were physically injured by the assault. **Table 1** provides further descriptive information about the participants at T1, as well as the participants who completed all four assessments. More detailed descriptions of the participants, the crime characteristics and participants’ emotions during the assault have been presented elsewhere (Johansen et al., 2007, 2008).

**Table 1:** Descriptive information for participants at T1 (*n* = 143) and those participating at all time points (*n* = 43).

	Sample at T1 (*n* = 143)		Sample responding at all time points (*n* = 43)		Sign. diff. dropouts^a^	
	*n*	%	*n*	%	χ^2^	*p*-value
*Gender*					0.11	0.74
Male	114	79.7%	35	81.4		
Female	29	20.3%	8	18.6		
*Marital status^b^*					0.51	0.77
Married/registered partner	25	17.6%	9	20.9%		
Single	101	71.1%	29	67.4%		
Divorced/separated	16	11.3%	5	11.6%		
*Education^b^*					12.10	0.02
Elementary school	11	7.7%	2	4.7%		
Intermediate-level education	50	35.2%	12	27.9%		
Upper-secondary education	31	21.8%	5	11.6%		
Higher education, up to 4 years	38	26.8%	18	41.9%		
Higher education, more than 4 years	12	8.5%	6	14.0%		
*Unemployed*					1.10	0.30
Yes	16	11.2%	3	7.0%		
No	127	88.8%	40	93%		
*Prior experience of violence*					0.35	0.56
Yes	63	47.7%	18	43.9%		
No	69	52.3%	23	56.1%		
*Violence category^c^*					0.33	0.56
Assault	45	31.5	15	34.9		
Inflicting bodily harm	98	68.5%	28	65.1%		
*Victim’s perception of threat level*					0.38	0.95
Felt life was at risk	50	42.4%	16	42.1%		
Fear of severe physical injury	25	21.2%	9	23.7%		
Understood danger only afterward	15	12.7%	4	10.5%		
Did not perceive as dangerous	28	23.7%	9	23.7%		

*^a^Significance test of the difference between the sample who participated at all time points (*n* = 43) and dropouts at any time point (*n* = 100). ^b^Information is missing for one participant. ^c^The injuries of each participant were classified into these legal categories at T1 in cooperation with the police and in accordance with a judgment based on the level of physical injury and the intention of the perpetrator to cause harm (where physical injury is the most important criterion) [The Norwegian General Civil Penal Code (Straffeloven) §§228 and 229] (Andenæs, 2004). The assault category includes less serious physical injuries, often combined with threats of more severe physical injury. The victims of inflicted bodily harm include people with more serious physical injuries, ranging from near-fatal injuries to bone fractures or other substantial damage.*

No significant differences were found between participants responding at all time points (*n* = 43) and dropouts at any time (*n* = 100) in terms of age, gender, prior experience of violence, physical injury, marital status, employment, perceived life threat, alcohol consumption, self-efficacy, or posttraumatic stress symptoms at T1. The participants responding at all time points did, however, have a higher mean education than the dropouts [*M*_diff_ = 0.57, *t*(140) = 2.83, *p* = 0.005].

### Assessment

#### Posttraumatic Stress Symptoms

Posttraumatic stress symptoms were assessed using The IES (Weiss and Marmar, 1997), a 22-item self-report questionnaire assessing current levels of intrusion, avoidance, and hyperarousal associated with the experience of a particular traumatic event. The items are scored on a 4-point scale (0 = not at all, 1 = rarely, 3 = sometimes, and 5 = often). The level of overall posttraumatic stress symptoms is reported as the mean of all 22 items, with a higher score representing more severe posttraumatic stress symptoms. Unlike a previous version of the IES (IES-15), there is no generally accepted diagnostic cutoff score for diagnosing PTSD with the version of the IES used in the present study (Creamer et al., 2003).

#### Perceived Self-efficacy

The Generalized Self-Efficacy Scale (GSE scale) (Schwarzer and Jerusalem, 1995) was used to measure self-perceived GSE. The GSE scale is a 10-item self-report scale with adequate psychometric properties (Schwarzer, 1993; Leganger et al., 2000; Johansen et al., 2013). The GSE scale assesses the respondent’s belief in his or her ability to adequately respond to novel or difficult situations and to cope with a large variety of stressors, and is scored on a 4-point scale from 1 (not at all true) to 4 (exactly true). The total GSE score is the mean score across all 10 items, with higher scores representing higher self-efficacy.

### Analyses

Attrition analyses were performed with Student’s *t*-tests and Pearson’s chi square tests. Stability in self-efficacy and posttraumatic stress symptoms were assessed with Pearson’s correlations and mixed-effects models using a restricted maximum likelihood (REML) estimation in which all available data without any imputations were used. The GSE and IES scores were transformed to *Z*-values across the four time points, with time defined as a nominal variable. One participant had extreme scores, and the data were therefore re-analyzed without this participant to check for undue influence (see Supplementary Material).

Whether self-efficacy predicted posttraumatic stress symptoms (at time point *t*) over and above prior posttraumatic stress symptoms (at time point *t* - 1) was assessed with a series of autoregressive cross-lagged models where the IES and GSE scores at a given time point (*t*) were simultaneously regressed on the immediately preceding time point (*t* - 1). These analyses also modeled the opposite relationship: whether posttraumatic stress symptoms (at time point *t*) predicted self-efficacy over and above prior self-efficacy (at time point *t* - 1). The models included gender, age and education as covariates because we considered these predictors for GSE and IES at T1.

All autoregressive models were analyzed with the full information maximum likelihood method (FIML) in SPSS AMOS (Schafer and Graham, 2002). The means and intercepts were estimated from the available raw data on a case-wise basis. Consequently, all participants who had participated at least once were included. The overall fit of the models was assessed with χ^2^ statistics with degrees of freedom and *p*-values, root mean square error of approximation (RMSEA) with 90% confidence intervals (CIs) and *p*-values, comparative fit index (CFI), and Tucker-Lewis index (TLI) (Browne and Cudeck, 1992; Hu and Bentler, 1999). The cutoff for acceptable model fit has been suggested to be 0.95 or above for CFI and TLI and from 0.06 to 0.08 or less for RMSEA (Browne and Cudeck, 1992; Hu and Bentler, 1999).

An unconstrained cross-lagged model (A) was compared for best fit with three more constrained models: model B, in which all cross-lagged paths between the GSE and IES were constrained to 0; model C, in which the paths from the prior GSE to later IES were set to 0; and model D, in which the paths from the prior GSE to later IES were set to be similar to the paths from the IES to GSE.

## Results

Our first analysis investigated changes in posttraumatic stress symptoms over time. Univariate mixed-effects analyses showed that the level of IES scores (**Table 2**) changed significantly over time [*F*(224.8) = 15.8, *p* < 0.001], with scores at T1, T2, and T3 being higher than scores at T4 (*b*_z-diff_ = 0.65, 0.46, and 0.42, respectively, all with *p* < 0.001). The IES scores at each assessment were highly correlated with the IES scores at all other time points (correlations ranged from 0.53 to 0.85, **Table 2**).

**Table 2:** Descriptive statistics and correlation matrix between posttraumatic stress symptoms and general self-efficacy (GSE) over time.

	Mean	*SD*	A	IES T2	IES T3	IES T4	GSE T1	GSE T2	GSE T3	GSE T4
IES T1	1.7	1.2	0.95	0.84^a^	0.65^b^	0.53^c^	-0.49^d^	-0.49^a^	-0.44^b^	-0.38^c^
IES T2	1.5	1.2	0.95		0.74^e^	0.65^f^	-0.45^a^	-0.45^a^	-0.48^e^	-0.51^f^
IES T3	1.4	1.3	0.95			0.85^f^	-0.25^b^	-0.29^e^	-0.47^b^	-0.46^f^
IES T4	0.9	1.1	0.96				-0.60^c^	-0.55^f^	-0.62^f^	-0.69^c^
GSE T1	3.2	0.5	0.89					0.79^a^	0.71^b^	0.76^c^
GSE T2	3.2	0.5	0.92						0.74^e^	0.67^f^
GSE T3	3.2	0.7	0.95							0.67^f^
GSE T4	3.3	0.7	0.96							

*Descriptive statistics (mean, *SD* and Cronbach’s alpha) and Pearson’s correlations between posttraumatic stress symptoms and GSE. All correlations are significant at *p* < 0.001. GSE, self-perceived GSE as measured by the Generalized Self-Efficacy Scale; IES, posttraumatic stress symptoms as measured by the Impact of Event Scale–22; T1, within 4 months after the assault; T2, 3 months after T1; T3, 12 months after T1; T4, 8 years after T1. ^a^*n* = 94; ^b^*n* = 73; ^c^*n* = 47; ^d^*n* = 143; ^e^*n* = 70; ^f^*n* = 44.*

**FIGURE 2:**
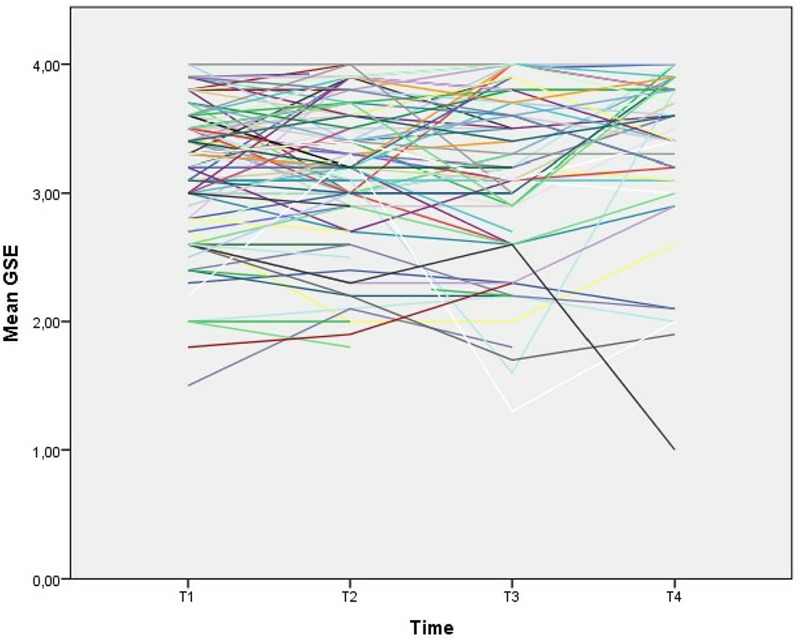
Individual levels of self-efficacy over time. Individuals’ mean levels of self-efficacy were measured with the Generalized Self-Efficacy Scale at each assessment point.

Our second analysis investigated the level and the stability of participants’ self-efficacy. GSE scores at all time points were highly correlated (between 0.67 and 0.79, **Table 2**). Many of the participants reported high levels of GSE, and there was a tendency for a ceiling effect. There were, however, also substantial individual differences in the change in GSE scores over time (**Figure 2**), although the GSE scores were, on average, stable across all four measurements. The main effect of time indicated that the changes in the mean GSE scores from T1 to T4 were not statistically significant [*F*(233.4) = 1.71, *p* = 0.17]. The highest levels of GSE were assessed at T4, but the level of GSE at T3 only was significantly lower than that at T4 (*b*_z-diff_ = -0.23, *p* = 0.03).

GSE and posttraumatic stress symptoms scores at all assessments were significantly negatively correlated, both concurrently (*r* = -0.45 to -0.69) and longitudinally (*r* = -0.25 to -0.62) (**Table 2**), with a tendency for posttraumatic stress symptoms at the last assessment having the highest correlation to self-efficacy at all time points (*r* = -0.55 to -0.69).

In our last analysis, we modeled the relationship between self-efficacy and posttraumatic stress symptoms across time in a series of autoregressive cross-lagged models. The unconstrained model had adequate model fits: χ^2^ = 56.38; *df* = 30; *p* ≤ 0.002; RMSEA = 0.08; 90% CI = 0.05–0.11; *p*-close = 0.07; CFI = 0.95; and TLI = 0.89 (**Table 3**). The RMSEA and TLI values were at or below the recommended cutoff values for model fit. However, these parameters are prone to over-reject true population models for small samples such as ours (Browne and Cudeck, 1992; Hu and Bentler, 1999). CFI was within an acceptable level of model fit.

**Table 3:** Model fits and model comparisons.

Model	χ^2^	*df*	*P* of χ^2^	*p* of χ^2^	CFI	NFI	TLI	RMSEA	90% CI of RMSEA	*p*-close
Model A (unconstrained)	56.375	30	0.002		0.949	0.903	0.888	0.079	0.046–0.110	0.071
Model B (all cross-lagged paths = 0)	80.262	36	<0.001	0.001	0.914	0.862	0.843	0.093	0.066–0.120	0.007
Model C (GSE to IES = 0)	66.031	33	0.001	0.022	0.936	0.887	0.872	0.084	0.054–0.113	0.033
Model D (GSE to IES = IES to GSE)	60.812	33	0.002	0.218	0.946	0.896	0.892	0.077	0.046–0.107	0.075

*CFI, comparative fit index; GSE, self-perceived GSE as measured by the Generalized Self-Efficacy Scale; IES, posttraumatic stress symptoms as measured by the Impact of Event Scale–22; NFI, the Bentler-Bonett normed fit index; RMSEA, root mean square error of approximation; and TLI, Tucker-Lewis index.*

**FIGURE 3:**
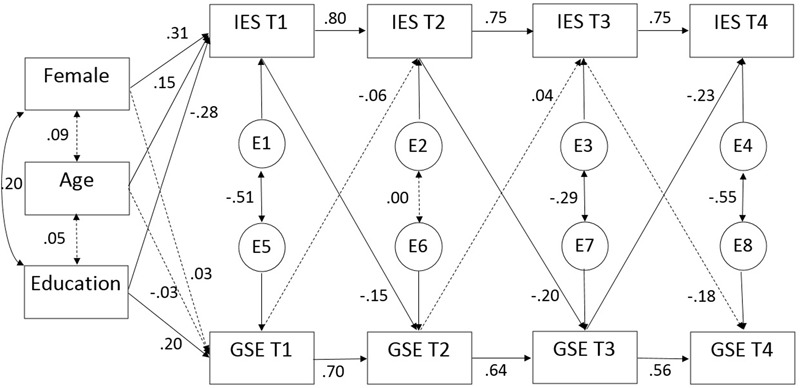
Cross-lagged SEM analyses of posttraumatic stress symptoms and self-efficacy across time. Autoregressive cross-lagged SEM analyses with standardized regression weights across four time points. Covariates were restricted to covary only with measures at T1. Insignificant relationships are indicated by dotted lines, whereas continuous lines indicate statistically significant (*p* < 0.05) relationships. GSE, self-perceived GSE as measured by the Generalized Self-Efficacy Scale; IES, posttraumatic stress symptoms as measured by the Impact of Event Scale-22.

The unconstrained model is presented in **Figure 3** and **Table 4**. The cross-lagged parameters from T1 to T2 and from T2 to T3 indicated a significant relationship between prior posttraumatic stress symptoms and later self-efficacy (IES-T1 → GSE-T2 *b*^∗^ = -0.15, *p* = 0.04; IES-T2 → GSE-T3 *b*^∗^ = -0.20, *p* = 0.02) but no significant relationship between prior self-efficacy and later posttraumatic stress symptoms. However, this result was reversed between T3 and T4, where prior self-efficacy was significantly related to later posttraumatic stress symptoms (GSE-T3 → IES-T4 *b*^∗^ = -0.23, *p* = 0.004), whereas posttraumatic stress symptoms at T3 were not significantly related to self-efficacy at T4 (*b*^∗^ = -0.18, *p* = 0.16). The critical ratios (CRs) between the concurrent cross-lagged estimates were all non-significant (CR of the cross-lagged estimates T1 to T2 = -0.52, *p* = 0.60; CR of the cross-lagged estimates T2 to T3 = 0.90, *p* = 0.37; CR of the cross-lagged estimates T3 to T4 = 1.83, *p* = 0.07). Thus, although only one of the cross-lagged estimates was significant at each time point (IES → GSE at T1 to T2 and T2 to T3, and GSE → IES at T3 to T4), the differences between the two concurrent estimates were not significantly different.

**Table 4:** Regression weights for cross-lagged SEM model of posttraumatic stress symptoms and self-efficacy over time.

	Estimate	Standard error	*p*-value
Gender → GSE T1	0.04	0.11	0.70
Gender → IES T1	0.94	0.23	<0.001
Age → GSE T1	0.00	0.00	0.69
Age → IES T1	0.02	0.01	0.05
Education → GSE T1	0.09	0.04	0.02
Education → IES T1	-0.30	0.08	<0.001
GSE T1 → GSE T2	0.74	0.08	<0.001
IES T1 → IES T2	0.78	0.06	<0.001
IES T1 → GSE T2	-0.07	0.03	0.04
GSE T1 → IES T2	-0.15	0.15	0.33
GSE T2 → GSE T3	0.80	0.11	<0.001
IES T2 → IES T3	0.81	0.10	<0.001
IES T2 → GSE T3	-0.11	0.05	0.02
GSE T2 → IES T3	0.09	0.21	0.68
GSE T3 → GSE T4	0.56	0.13	<0.001
IES T3 → IES T4	0.69	0.07	<0.001
IES T3 → GSE T4	-0.10	0.07	0.16
GSE T3 → IES T4	-0.41	0.14	0.004

*Unstandardized regression weights in the unconstrained cross-lagged SEM model (**Figure 3**). GSE, self-perceived GSE as measured by the Generalized Self-Efficacy Scale; IES, posttraumatic stress symptoms as measured by the Impact of Event Scale–22; T1, within 4 months after the assault; T2, 3 months after T1; T3, 12 months after T1; T4, 8 years after T1.*

The model (**Figure 3** and **Table 4**) indicated high consistency over time for both posttraumatic stress symptoms (standardized regression coefficients *b*^∗^ = 0.75 to 80, all *p* ≤ 0.001) and self-efficacy (*b*^∗^ = 0.56 to 0.70, all *p* ≤ 0.001).

Gender was significantly related to posttraumatic stress symptoms but not to self-efficacy at T1. Women reported a higher level of posttraumatic stress symptoms than men (*b*^∗^ = 0.31, *p* < 0.001) (**Table 5**). Age was significantly positively related to posttraumatic stress symptoms (*b*^∗^ = 0.15, *p* = 0.05) but not to self-efficacy at T1. Higher education was significantly related to lower posttraumatic stress symptoms (*b*^∗^ = -0.28, *p* < 0.001) and higher levels of self-efficacy (*b*^∗^ = 0.20, *p* = 0.02) at T1.

**Table 5:** Regression weights (unstandardized) for covariates in the autoregressive cross-lagged model.

	Estimate	Standard error	*p*-value
E1 ↔ E5	-0.30	0.06	<0.001
E2 ↔ E6	0.00	0.02	0.98
E3 ↔ E7	-0.11	0.05	0.02
E4 ↔ E8	-0.15	0.05	0.001
Gender ↔ Education	0.09	0.04	0.02
Gender ↔ Age	0.38	0.37	0.30
Age ↔ Education	0.64	1.04	0.54

*Unstandardized regression weights in unconstrained cross-lagged SEM model (**Figure 3**). GSE, self-perceived GSE as measured by the Generalized Self-Efficacy Scale; IES, posttraumatic stress symptoms as measured by the Impact of Event Scale–22.*

Constraining the model caused changes in model fit. The more constrained model B, with all cross-lagged paths being constrained to equal 0, had worse fit indices (**Table 3**). Thus, the cross-lagged paths contributed significantly to explaining the GSE and IES scores over time. Moreover, the constrained model C, with the paths from GSE to IES constrained to be equal to 0, showed a worse model fit (**Table 3**). This finding indicates that the paths from GSE to IES contributed significantly to the model fit in the unconstrained model. However, model D, in which the paths from GSE to IES were set as equal to the paths from IES to GSE, did not have significantly worse fit indices than the unconstrained original model (**Table 3**), thus indicating that the paths from GSE to IES may not be different from the paths from IES to GSE.

## Discussion

The present study examined the relationship between posttraumatic stress symptoms and self-efficacy in assault victims during an 8-year follow-up period. We hypothesized that higher levels of self-efficacy at the first assessment would be related to subsequently lower levels of posttraumatic stress symptoms.

We found levels of bivariate cross-sectional and longitudinal relationships between self-efficacy and posttraumatic stress symptoms that were similar to those reported in previous reviews (Luszczynska et al., 2009; Simmen-Janevska et al., 2012). However, the results are more complex when all the longitudinal aspects and possible reciprocal relationships are considered, and our hypothesis was only partly supported by these longitudinal models. We did not find evidence for an association between self-efficacy and subsequent posttraumatic stress symptoms in the first year after the assault. Higher levels of posttraumatic stress symptoms in the first 3 months after the assault were related to lower levels of self-efficacy at 3 and 12 months post-assault. However, from 12 months to 8 years after the assault, although self-efficacy was negatively associated with subsequent posttraumatic stress symptoms, no evidence was found for the reverse relation.

We were not able to replicate the findings from previous studies showing that self-efficacy is related to lower levels of posttraumatic stress symptoms during the first 12 months after a traumatic incident (Benight and Harper, 2002; Bosmans and van der Velden, 2015). The reason for these divergent findings may be that we investigated *generalized* self-efficacy, while these prior studies investigated trauma-related *coping* self-efficacy. However, our findings indicate that GSE may facilitate posttraumatic stress symptom recovery over a longer period, i.e., after 1 year. As expressed by several of our participants during the interviews, during the posttraumatic stress recovery process, it is important to first be aware of and accept one’s own reactions and symptoms; then, as a second step, one should choose strategies to combat these symptoms.

Our novel finding that posttraumatic stress symptoms in the first year after the assault are negatively related to subsequent self-efficacy indicates that cross-sectional relationships (Luszczynska et al., 2009; Simmen-Janevska et al., 2012) may be partially due to a reversed causality between self-efficacy and posttraumatic stress symptoms. Participants’ beliefs in their ability to succeed may have been shattered by the posttraumatic stress symptoms they experienced (Janoff-Bulman, 1992; Nygaard and Heir, 2012). There might also be processes specifically related to being a victim of an assault that are important for these processes. Compared to victims of non-intentional trauma (e.g., natural disasters or accidents), assault victims more often experience a chronic PTSD course (Santiago et al., 2013). In addition to the personal violation, assault victims must address a variety of stressors, such as insurance claims, pain, inability to work, difficult decisions about reporting the trauma to authorities and facing their attacker in court. The waiting time for the case to be processed in the juridical system can also cause substantial stress. The relatively slow recovery process from assault may thus be partly explained by the demands of such ongoing court processes (Osenbach et al., 2009). As one participant in the present study reported in an in-depth interview after 8 years, “I feel that the adverse experience consisted of the entire process, including the court process. Even though what occurred that night was actually the most important part, when I think back on it now, afterward, it feels like the entire first year was part of the experience.” Additionally, some of our victims reported symptom deterioration if the police closed their case or they lost in court. Over time, as the symptom levels decrease and the victims regain confidence in their ability to cope, the influence of self-efficacy on posttraumatic stress symptoms may increase. Our results are partially supported by a study of motor vehicle accident survivors, which reported an increase in self-efficacy levels only among those whose PTSD symptoms were significantly reduced during therapy (Cyniak-Cieciura et al., 2015).

Average self-efficacy was remarkably stable over time, while posttraumatic stress symptoms decreased. The stability of GSE in post-trauma populations is in line with findings from previous studies (Heinrichs et al., 2005; Johansen et al., 2007; Nygaard et al., 2016), and self-efficacy in general may be stable over time (Solomon et al., 1991; Heinrichs et al., 2005; Johansen et al., 2007; Simmen-Janevska et al., 2012) with a trait-like structure (Chen et al., 2001). The trait-like qualities of self-efficacy are corroborated by research on twins indicating that these trait-like qualities might be related to genetic factors (Waaktaar and Torgersen, 2013). Nevertheless, there were some differences in individual trajectories (**Figure 2**). Furthermore, posttraumatic stress symptoms appeared to have some effect on individual levels of GSE in the first year. Thus, there were also changes in individuals’ self-efficacy levels over time, which were, in part, related to posttraumatic stress symptoms. High levels of posttraumatic stress symptoms may be related to lower self-efficacy in the short term, while other processes related to trauma experiences work in the opposite direction, maintaining a similar mean level of self-efficacy at the group level. However, the present study was not able to disentangle these processes.

The possibility that posttraumatic stress symptoms may lower self-efficacy may have some clinical implications for the ideal time at which to treat stress symptoms. An increased risk of chronic PTSD 5 months post-trauma (Morina et al., 2014) emphasizes the importance of early prevention and treatment. Possible negative consequences of PTSD on self-efficacy in the first year post-trauma substantiate guidelines recommending treatment for PTSD that lasts more than 3 months (NICE, 2005) to accelerate the recovery process and avoid its negative impact on self-efficacy.

### Strengths and Limitations

The main strength of our study is the longitudinal design, which allowed cross-lagged statistics. Assessing posttraumatic stress symptoms and perceived self-efficacy at four time points over an 8-year period had not previously been performed. This study design offered the possibility of studying the effects of these variables in short, intermediate, and longer terms.

Another important strength of our study is the homogeneity of the potential traumatic event. All of the respondents were exposed to physical assault by a perpetrator other than a family member. The gender distribution at T1 was representative of exposure to a violent crime (other than domestic assault) in Norway (Steen and Hunskaar, 2004; Statistics Norway, 2009).

The cross-lagged statistical models allowed us to minimize the undesirable effect of confounders. It is, however, important to be aware of the possibility that other models may fit the data equally well (Blunch, 2013). While we compared the fit of the proposed cross-lagged model (A) with that of three other theoretically relevant models, future research may identify other models fitting the data equally well or better. Furthermore, to improve the validation of our study by minimizing recall bias, we performed the interviews at T1 as soon as possible after the event. We also knew the exact date of exposure and the elapsed time from the exposure to the interview for all participants.

The prime methodological limitation of this study is the small sample size; only 43 participants completed all the assessments over the full 8 years. Small sample sizes and high dropout levels are a common problem in longitudinal studies of assault victims (Shalev et al., 1998; Andrews et al., 2003; Brewin et al., 2003; Elklit and Brink, 2004). Fourteen of the 143 invited participants at T3 and 10 of the 97 invited participants at T4 could not be reached by mail due to unknown addresses. However, the respondents at all time points (*n* = 43) were comparable to the dropouts in all respects except the level of education. All information given by any participant at any time was included in the model analyses, thus reducing the dropout effect.

Both the IES and the GSE scale are among the most-used questionnaires for measuring posttraumatic stress symptoms and self-efficacy, and they have been found to be both reliable and valid measures (Schwarzer, 1993; Leganger et al., 2000; Creamer et al., 2003). An advantage of using self-reports at all time points is that their dimensional view provided information on relative changes in posttraumatic stress symptoms and self-efficacy over time.

There may be discrepancies between studies of GSE, such as the present study, and studies of CSE. Whereas GSE is the belief in one’s competence to tackle novel tasks and to cope with adversity over a broad range of stressful or challenging encounters, specific self-efficacy or CSE is constrained to the belief in one’s ability to cope with a particular task. Parts of some measures of trauma CSE may therefore be quite similar to posttraumatic stress cognitions and symptoms, just with the opposite sign. Finally, specific trauma-related beliefs about coping are more closely related to PTSD symptoms in the first months after the trauma, while the effect of GSE is more prominent over longer periods (Cyniak-Cieciura et al., 2015).

We have no nuanced information about the pharmacological and/or psychological treatments the participants may have received, their duration, or their effects. Victims exposed to non-domestic violence are typically not systematically offered follow-up treatment through the Norwegian public health service.

## Conclusion

The relationship between GSE and posttraumatic stress symptoms seems to be complex; these constructs can affect each other over the course of many years. From a long-term perspective, strengthening GSE can have effects on posttraumatic stress symptoms in trauma survivors, assuming that GSE is actually responsive to intervention. Further research is needed to determine whether GSE can be altered externally over time. Our study shows that many assault victims have high levels of posttraumatic stress symptoms after as long as 8 years. Further research is necessary to identify measures that can help this group and determine how effective interventions can be performed.

## Author Contributions

EN analyzed and interpreted the data and drafted the manuscript. VJ designed the study and acquired the data. VJ, JS, AH, and TH contributed substantially to the interpretation of the data and drafting of the manuscript, and they revised the manuscript critically for important intellectual content. All authors have approved the final version of the manuscript and have agreed to be accountable for all aspects of the work in ensuring that questions related to the accuracy or integrity of any part of the work are appropriately investigated and resolved.

## Conflict of Interest Statement

The authors declare that the research was conducted in the absence of any commercial or financial relationships that could be construed as a potential conflict of interest.

## Abbreviations

CSE: coping self-efficacy
GSE: general self-efficacy
IES: Impact of Event Scale–22
PTSD: posttraumatic stress disorder
