# Supplementary material for: Longitudinal Relationship between Self-efficacy and Posttraumatic Stress Symptoms 8 Years after a Violent Assault: An Autoregressive Cross-Lagged Model
Source: Front Psychol. 2017 Jun 1;8:913. doi: 10.3389/fpsyg.2017.00913 (PMC5452477; doi:10.3389/fpsyg.2017.00913)
Supplement: Supplementary file 1 [file Table_1.DOCX]

# Supplementary material

# Longitudinal Relationship Between Self-Efficacy and Posttraumatic Stress Symptoms 8 Years After a Violent Assault: An Autoregressive Cross-lagged Model

Egil Nygaard*, Venke A. Johansen, Johan Siqveland, Ajmal Hussain, and Trond Heir

*** Correspondence**:

Egil Nygaard

[egilny@psykologi.uio.no](mailto:egilny@psykologi.uio.no)

## Analyses without the outlier

Distributional analyses revealed one participant with extreme values for all the continuous variables (posttraumatic stress symptoms, general self-efficacy, and age) (> 3 *SD* from mean), which, as observed from the histograms, were outside a normal distributional curve. Therefore, all analyses were re-performed without this participant to determine whether the distributional skewness had unduly influenced the results. Both bivariate analyses (Table A.1) and the results of the unconstrained autoregressive cross-lagged model (Tables A.2 and A.3) without this participant were nearly identical to the same tests when all the participants were included. The fit indices were also similar, with the unconstrained model having the following model fits: χ^2^ = 50.11; *df* = 30; *p* ≤ .012; root mean square error of approximation (RMSEA) = .07; 90% CI = .03–.10; *p*-close = .17; comparative fit index (CFI) = .96; and Tucker-Lewis index (TLI) = .91. The mixed-effects model of change in posttraumatic stress symptoms produced nearly identical results when this participant was excluded and when all participants were included (*F*(222.5) = 16.9, *p* < .001), with scores at T1, T2 and T3 lower than those at T4 (*b*_z-diff_ = 0.67, 0.47 and 0.44, respectively, all with *p* < .001). However, the mixed-effects model of stability in self-efficacy over the four time points showed the increase in GSE more clearly from the earlier time points to T4 when the participant with extreme scores was excluded. The total effect of time was significant (*F*(228.7) = 3.04, *p* = .03), and the mean levels of self-efficacy at T1 (*b*_z-diff_ = -.25, *p* = .01), T2 (*b*_z-diff_ = -.20, *p* = .05) and T3 (*b*_z-diff_ = -.30, *p* = .05) were all lower than those at T4.

Table A.1

Descriptive statistics and correlation matrix between posttraumatic stress symptoms and general self-efficacy across time **without the extreme outlier values**

|  | Mean | *SD* | α | IES T2 | IES T3 | IES T4 | GSE T1 | GSE T2 | GSE T3 | GSE T4 |
| --- | --- | --- | --- | --- | --- | --- | --- | --- | --- | --- |
| IES T1 | 1.7 | 1.2 | .95 | .84^a^ | .65^b^ | .53^c^ | -.49^d^ | -.49^a^ | -.44^b^ | -.41^c^ |
| IES T2 | 1.5 | 1.2 | .95 |  | .74^e^ | .63^f^ | -.44^a^ | -.43^a^ | -.47^e^ | -.46^f^ |
| IES T3 | 1.4 | 1.3 | .96 |  |  | .85^f^ | -.24^b^ | -.28^e^ | -.46^b^ | -.46^f^ |
| IES T4 | 0.9 | 1.1 | .95 |  |  |  | -.58^c^ | -.52^f^ | -.61^f^ | -.66^c^ |
| GSE T1 | 3.2 | 0.5 | .89 |  |  |  |  | .79^a^ | .71^b^ | .78^c^ |
| GSE T2 | 3.2 | 0.5 | .91 |  |  |  |  |  | .73^e^ | .64^f^ |
| GSE T3 | 3.2 | 0.7 | .95 |  |  |  |  |  |  | .71^f^ |
| GSE T4 | 3.4 | 0.6 | .95 |  |  |  |  |  |  |  |

Note: Descriptive (mean, SD and Cronbach’s alpha) and Pearson’s correlations between posttraumatic stress symptoms and general self-efficacy. All correlations are highly significant (*p* < .001).

GSE = Self-perceived general self-efficacy as measured by the Generalized Self-Efficacy Scale; IES = Posttraumatic stress symptoms as measured by the Impact of Event Scale–22

^a^ *n* = 93; ^b^ *n* = 72; ^c^ *n* = 46; ^d^ *n* = 142; ^e^ *n* = 69; ^f^ *n* = 43

Table A.2

Regression weights for the cross-lagged SEM model of posttraumatic stress symptoms and self-efficacy across time **without the extreme outlier values**

|  | Estimate | Standard error | *p*-value |
| --- | --- | --- | --- |
| Gender → GSE T1 | 0.04 | 0.11 | .73 |
| Gender → IES T1 | 0.94 | 0.24 | < .001 |
| Age → GSE T1 | 0.00 | 0.00 | .83 |
| Age → IES T1 | 0.02 | 0.01 | .06 |
| Education → GSE T1 | 0.09 | 0.04 | .02 |
| Education → IES T1 | -0.30 | 0.08 | < .001 |
| GSE T1 → GSE T2 | 0.73 | 0.08 | < .001 |
| IES T1 → IES T2 | 0.78 | 0.06 | < .001 |
| IES T1 → GSE T2 | -0.07 | 0.03 | .03 |
| GSE T1 → IES T2 | -0.11 | 0.15 | .45 |
| GSE T2 → GSE T3 | 0.82 | 0.10 | < .001 |
| IES T2 → IES T3 | 0.82 | 0.10 | < .001 |
| IES T2 → GSE T3 | -0.12 | 0.05 | .02 |
| GSE T2 → IES T3 | 0.07 | 0.22 | .75 |
| GSE T3 → GSE T4 | 0.53 | 0.11 | < .001 |
| IES T3 → IES T4 | 0.68 | 0.07 | < .001 |
| IES T3 → GSE T4 | -0.07 | 0.06 | .20 |
| GSE T3 → IES T4 | -0.39 | 0.14 | .004 |

Note: Unstandardized regression weights in the cross-lagged SEM model (Figure 3) (*n* = 142).

GSE = Self-perceived general self-efficacy measured by the Generalized Self-Efficacy Scale; IES = Posttraumatic stress symptoms measured by the Impact of Event Scale–22

Table A.3

Regression weights (unstandardized) for covariates in the autoregressive cross-lagged model **without the extreme outlier values**

|  | Estimate | Standard error | *p*-value |
| --- | --- | --- | --- |
| E1 ↔ E5 | -0.27 | 0.05 | < .001 |
| E2 ↔ E6 | 0.01 | 0.02 | .73 |
| E3 ↔ E7 | -0.11 | 0.05 | .02 |
| E4 ↔ E8 | -0.10 | 0.04 | .02 |
| Gender ↔ Education | 0.09 | 0.04 | .02 |
| Gender ↔ Age | 0.42 | 0.37 | .26 |
| Age ↔ Education | 0.80 | 1.04 | .44 |

Note: Unstandardized regression weights in the cross-lagged SEM model (Figure 3) (*n* = 142).
